# Psychopharmacology of Pediatric Anxiety Disorders: A Narrative Review

**DOI:** 10.7759/cureus.5487

**Published:** 2019-08-26

**Authors:** Afshan N Amray, Khurram Munir, Nusrat Jahan, Fatima B Motiwala, Sadiq Naveed

**Affiliations:** 1 Medicine, Dow Medical College and Civil Hospital Karachi, Dow University of Health Sciences, Karachi, PAK; 2 Physiology, Sheikh Zayed Medical College and Hospital, Rahim Yar Khan, PAK; 3 Internal Medicine, Rush University Medical Center, Chicago, USA; 4 Psychiatry, Texas Tech University Health Science Center, Midland, USA; 5 Psychiatry, University of Kansas Medical Center, Kansas City, USA

**Keywords:** anxiety, youth, psychopharmacology

## Abstract

Anxiety disorders are common among children and adolescents; almost one-third of this population has an anxiety disorder. The most common anxiety disorders in this population are specific phobia (19.3%), social anxiety disorder/ social phobia (9.1 %), and separation anxiety disorder (7.6 %). Pediatric anxiety disorders are often associated with poor psychosocial functioning, academic underachievement, learning difficulties, substance abuse, relationship problems, and suicide behaviors. Psychotherapy, particularly cognitive behavioral therapy as a stand-alone treatment or in combination with medication, is found to be efficacious in the treatment of various anxiety disorders. The early recognition and treatment of anxiety disorders result in better long-term outcomes in children and adolescents. This article summarizes the evidence-based pharmacologic treatments for anxiety disorders in youth, including social anxiety disorder generalized anxiety disorder, separation anxiety disorder, and panic disorder.

## Introduction and background

Anxiety disorders are the most common psychiatric disorders in adolescents and young adults with a lifetime prevalence of up to 30%. In a national survey, lifetime prevalence rates for adolescents were 31.9 % for any anxiety disorder, 19.3 % for specific phobia, 9.1 % for social anxiety disorder (SAD), 7.6 % for separation anxiety disorder, 2.3 % for panic disorder, 2.4 % for agoraphobia, and 2.2 % for generalized anxiety disorder (GAD). In adolescents, anxiety disorders are more prevalent among girls than boys, with an estimated ratio of 2:1 [1]. Anxiety disorders have an early onset age among children and adolescents with specific phobia and separation anxiety disorders occurring during childhood at age 6-8, while the age of onset for GAD, agoraphobia, and panic disorder is usually in adolescence [2-3].

Anxiety disorders usually have a chronic and persistent course as suggested by the prevalence rates throughout the life span [4]. The period prevalence rates for 6 months or 1 year are comparable to lifetime estimates, indicating a low remission rate for anxiety disorders and a higher risk of recurrence [4-5]. The presence of childhood anxiety disorders increases the risk of anxiety disorders in adulthood, suggesting a chronic and persistent course [5]. The trajectory of anxiety disorders suggests that psychosocial and biological determinants, such as lower parental warmth, parenting psychopathology, anxiety, and depressive symptoms, familial health problem, family dysfunction, poverty, and child abuse are associated with higher rates of persistence of anxiety disorders [6].

Anxiety disorders are associated with significant functional impairment and the development of the co-morbid psychiatric disorder in adulthood. They also result in a higher risk of cognitive impairment, academic under-achievement, and social dysfunction [7]. Moreover, childhood anxiety disorders are associated with homotypic continuity of anxiety disorders, depressive disorder, suicidal behaviors, nicotine and alcohol dependence, and other substance use disorders [8]. The data from The Great Smoky Mountain Study showed the poor long-term outcome of several childhood anxiety disorders. Childhood overanxious disorder predicted the development of an overanxious disorder, panic attacks, and depression and conduct disorders in adolescents [9]. In this longitudinal study of multiple anxiety disorders in childhood, 20% of the subjects continued to meet criteria for anxiety disorders as young adults and almost all of the initial subjects exhibited poor functioning across domains. A childhood diagnosis of GAD / and overanxious disorder was associated with the poorest functional outcome in early adulthood [10]. The burden of anxiety disorders and the associated functional impairment necessitate early recognition and treatment of anxiety disorders.

In this review article, we have included the pharmacological treatment for the disorders that are currently recognized under the umbrella of anxiety disorder in the current Diagnostic and Statistical Manual of Mental Disorders (DSM)- 5. The existing literature has been assessed for the efficacy and safety of existing treatment options for SAD, GAD, panic disorder, and separation anxiety disorder. Selective serotonin reuptake inhibitors (SSRIs) are frequently prescribed for the treatment of anxiety disorders in youth. Only a few medications are approved by the Food and Drug Administration (FDA) to treat anxiety disorders in children and adolescents. Table 1 lists these medications that have been FDA approved for the treatment of both obsessive compulsive disorder (OCD) and non-OCD anxiety disorders.

**Table 1:** FDA Approved Medications in Pediatric Anxiety Disorders FDA: Food and Drug Administration; OCD: Obsessive Compulsive Disorder

Medications	OCD	Panic Disorder	Social Anxiety Disorder	Generalized Anxiety Disorder
Clomipramine	≥10			
Duloxetine				≥7
Fluoxetine	≥7			
Fluvoxamine	≥8			
Sertraline	≥6			

A combination of treatments is often more efficacious. The results of the Child/ Adolescent Multimodal Study (CAMS) show that a combination of CBT and sertraline (80.7%) was better than CBT alone (59.7%) or sertraline alone ( 54.9%) to treat non-OCD anxiety disorder (GAD, social phobia, separation anxiety) [11].

## Review

Methods

In August 2018, the PubMed and Scopus were systematically searched for relevant publications focused on pharmacological treatments of SAD, GAD, panic disorder and separation anxiety disorder in humans. Two independent reviewers performed the manual search of references and relevant articles for included studies. Search results from these databases were imported to Endnote ×7 (Thompson Reuter, CA, USA) to remove any duplicates. Two independent reviewers performed title and abstract screening (when available) followed by the full-text screening of the articles by selecting case reports, case series, open-label trials, and randomized controlled trials (RCTs). The abstract-only articles, conference papers without original data, reviews, theses, posters, book chapters, editorials, letters, and commentaries were excluded. No restrictions on language, country, publication year, age, gender, or ethnicity of patients were applied. This article provides a summary of the evidence-based pharmacologic treatments for different anxiety disorders in children and adolescents based on the available data. 

Social anxiety disorder

1. Sertraline

In an open-label trial of 8 weeks, the efficacy and safety of sertraline were assessed among 14 participants, ages 10-17 (eight boys and six girls), with SAD. Sertraline was initiated at 50 mg/day with dinner, and it was titrated at 50-mg increments every other week to reach a maximum dose of 200 mg/day by week 6, depending on the clinical response and tolerability. At the end of the study, the doses were 200 mg/day in one, 150 mg/day in five, 125 mg/day in one, and 100 mg/day in four participants. The average daily dose of sertraline was 123.21 ± 37.29 mg/day at the end of the study. The Clinical Global Impression-Improvement scale (CGI-I) was used to measure improvement relative to baseline and the Clinical Global Impression - Severity scale (CGI-S) was used to measure severity. The Social Phobia and Anxiety Inventory for Children and a standardized behavioral avoidance test were used to assess for anxiety. By the end of the eight weeks, the independent evaluator reported that 5/14 (36%) subjects were classified as treatment responders or partial responders 4/14 (29%). However, psychiatrists rated 3/14 (21%) subjects as treatment responders and 7/14 (50%) as partial responders. The most common side effects were drowsiness, nausea, diarrhea, jitteriness, restlessness, headache, trouble sleeping, aggression, and skin rash in descending order of frequency. Most of these side effects were transient and resolved with a reduction in the dose of sertraline. Sertraline was discontinued in one patient due to severe gastrointestinal side effects [12].

2. Escitalopram

The efficacy and safety of escitalopram were assessed in a 12-week open-label trial among 20 youth (10-17 years old) with SAD. Escitalopram was started at 5 mg/day in the first week, and then it was increased to 10 mg/day in the second week. The dose was maximized to 20 mg/day at 5-mg increments depending on the clinical response and tolerability. At the end of the study, 12 (60%) patients were taking 10 mg/day of escitalopram, four (20%) were taking 15 mg/day, and another four (20%) were taking 20 mg/day. The clinical efficacy was assessed by a change in CGI-I scores indicating that 13 of 20 (65%) patients achieved a score of 2 indicating a response to treatment. All secondary outcomes and quality of life measures showed improvements from baseline to week twelve, with effect sizes ranging from 0.9 to 1.9. The average daily dose of escitalopram over the 12-week open-label was 12.65 ± 2.08 and at the endpoint was 13 ± 4.1 mg. Somnolence, insomnia, flu symptoms, and appetite change were frequent, but transient side effects. Tremors were reported in one patient and were resolved on discontinuation [13].

3. Paroxetine

In a 16-week long, double-blind RCT, the efficacy and tolerability of paroxetine were evaluated for SAD among 322 children and adolescents (ages 8-17 years old). Participants were randomized to receive paroxetine (10-50 mg/day) or placebo. Paroxetine was started at 10 mg/day and was titrated to a maximum dose of 50 mg/day at 10 mg-increments per week. The average dose for paroxetine was 32.6 mg/day for all patients (26.5 mg/day for children and 35.0 mg/day for adolescents) at the end of the study. The CGI-I was used to assess for efficacy at the endpoint, a score of ≤ 2 indicating a response. Safety was evaluated at each visit by monitoring vital signs and adverse effects. About 95.6% of participants were categorized as high risk. Further breakdown yielded 45.1% were “moderately ill”, another 38.5% were “markedly ill”, and 12% were “severely ill” by using CGI-S with higher comorbidity among the paroxetine group. About 77.6% of participants receiving paroxetine were defined as responders compared to 38.3% receiving a placebo. The clinical benefits of paroxetine were noticeable within the first four weeks of treatment. At the endpoint, participants receiving paroxetine (47.2%) were more likely to achieve remission compared to placebo (13.3%). The most common adverse effects were insomnia, decreased appetite, and vomiting. These side effects were mild and transient. Behavioral side effects included nervousness, hyperkinesia, asthenia, hostility, somnolence, insomnia, agitation, manic reaction, and emotional liability. Four patients receiving paroxetine reported suicidal ideations with one patient displaying self-harm behaviors [14].

4. Tandospirone

Huang and colleagues examined the efficacy and safety of tandospirone compared to sertraline in an open-label trial of eight weeks among 71 adolescents (ages 14-21 years) with SAD. Tandospirone is a partial agonist of serotonin _1A_ receptor with anxiolytic properties. Tandospirone was started at a fixed dose of 10 mg twice daily for the first week with a gradual increase to three times/day. The maintenance dose was between 20 to 60 mg/day depending on the efficacy and safety. Sertraline was initiated at 25 mg/day during the first week and was titrated to 50 mg/day in the second week. The mean dose was 35.14±7.75 mg for tandospirone and 89.58±30.47 mg for sertraline at the end of the study with a maintenance dose of 25-200 mg/day. The efficacy was assessed by Hamilton Anxiety (HAM)-A scores and change from baseline in CGI-I scores. Vital signs and adverse events were assessed at baseline and at weeks two, four, six, and eight by using the Treatment-Emergent Scale. The response rates were 48.6% for tandospirone and 55.6% for sertraline on the CGI-I scale. By using the HAM-A scale, the rate of response was 37.1% for tandospirone and 41.7% for sertraline. The most common side effect was drowsiness (11.4%), fatigue, and decreased appetite (8.6%) for tandospirone and decreased appetite (13.9%) followed by nausea (11.1%) for sertraline. The study demonstrates the efficacy and safety of tandospirone for adolescents, showing it to be as effective as sertraline for treating SAD [15].

5. Citalopram

Chavira and colleague assessed the efficacy of citalopram in a 12-week long, open-label trial along with psychoeducational treatment. All 12 participants (ages 8-17 years old) received 12 weeks of citalopram treatment and eight brief CBT-oriented counseling sessions (15 minutes each). Of these 12 participants, two participants dropped out due to nausea, lightheadedness, or concentration problems. Citalopram was started at 10 mg/day, and it was increased at 10-mg increments with a maximum dose of 40 mg/day over 12 weeks. About 83% of the participants were reported as being very much improved (41.7%) and much improved (41.7%) on the CGI-I scale. The treatment was well-tolerated without any side effects during the course of study [16].

6. Cannabidiol

A double-blind randomized trial tracked the effects of a simulation public speaking test (SPST) among 12 healthy control participants and 24 patients with SAD who received a single dose of Cannabidiol (CBD) or placebo before the test. After careful screening, a total of 24 SAD subjects who never received any treatment for SAD were randomized to either CBD 600 mg (n=12) and placebo (n=12). The control subjects were assigned to perform the SPST without any medical intervention. CBD was given before the test. Psychological assessment was conducted by using the Visual Analogue Mood Scale (VAMS) and Negative Self-Statement scale (SSPS-N), and physiological measures including blood pressure, heart rate, and skin conductance. This study reported that pretreatment with CBD resulted in improvement in anxiety, cognitive impairment, and discomfort in their speech performance. It also resulted in a significant reduction of alertness in their anticipatory speech, compared to the placebo group. There was no significant difference observed among the healthy control participants [17].

7. Several Serotonergic Agents

Mancini and colleagues reported the efficacy of different serotonergic agents for social phobia among seven participants. The age range for these participants was 7 to 18 years (six female and one male) with a mean age of 14.4 years. Of the seven patients, four had comorbid anxiety and mood disorder. The mean age of onset for social phobia was six years. These patients were treated with paroxetine (n=5), sertraline (n=1), and nefazodone (n=1). The response to treatment was assessed by retrospective chart review performed by treating physicians. All patients responded to serotonergic agents, and their anxiety improved with minimal side effects. The most common side effects reported by three participants were transient diarrhea, minor visual accommodation difficulty, and mild somnolence. The dose range was 5-80 mg for paroxetine, 175 mg for sertraline, and 400 mg for nefazodone [18].

8. Fluoxetine

In an RCT, the effectiveness of fluoxetine, placebo, and social effectiveness therapy (SET-C) was studied among children and adolescents (age 7-17 years) with social phobias [19]. In this study, 139 participants were randomized into SET-C (n=52), fluoxetine (n=33), or placebo group (n=33). Fifteen participants dropped out of the study after randomization by eight weeks, and two were excluded due to noncompliance. SET-C therapy consisted of social skills training, peer generalization experiences, and in vivo exposure [20]. Fluoxetine was prescribed following this titration schedule: weeks one and two, 10 mg per day; weeks three and four, 20 mg per day; and weeks five and six, 30 mg per day. The dose was increased to 40 mg at week six and was continued for the duration of the study (through week 12). The outcome measures were evaluated using self-reports of symptoms, parent ratings, independent evaluator ratings, and behavioral assessment. At study endpoint, 79% of participants receiving SET-C therapy were ranked as responders compared to 36.4% and 6.3% for fluoxetine and placebo group, respectively. The remission rates were 53% for SET-C, 21.2% for fluoxetine and 3.1% for the placebo group. The response for SECT-C and fluoxetine was assessed at one year for responders during the RCT. At one-year follow-up, almost all participants continued to report improvement with one-year relapse rates of 17% for fluoxetine and 10.3% for SET-C [19].

9. Venlafaxine Extended-Release (ER)

A 16-week placebo-controlled RCT evaluated the efficacy, safety, and tolerability of venlafaxine ER in 293 youth aged 8-17 years with SAD. Participants were randomized at baseline visit to venlafaxine ER or placebo in a double-blind manner. Venlafaxine ER was started at 37.5 mg /day and titrated up to 75 mg/day depending upon weight and clinical symptoms. The efficacy was assessed by Social Anxiety Scale-child or adolescent version (SAS-CA), and responder assessment was performed by CGI-I scores at each visit. Safety was assessed by measuring weight, heart rate, temperature, and electrocardiogram (EKG) during the visits. About 35% of participants dropped out of the study due to lack of efficacy and 4% due to the adverse effects. The average daily dose of venlafaxine ER during the active treatment phase was 141.5±47.5 mg for > 0 days of treatment among 140 participants and 155±39.0 mg for > 112 days of treatment among 54 participants. The average daily dose was given at 2.6 mg/kg to 3 mg/kg.

A statistically significant benefit was observed for venlafaxine ER on the SAS-CA compared to placebo with a number needed to treat of five. About 56 % of venlafaxine ER was scored 1 (very much improved) or 2 (much improved) on the CGI-I scale compared to 37% among the placebo group. About 90% of participants reported treatment-emergent adverse effects which were mild-to-moderate in severity. These side effects resolve over the course of study with continued treatment. The most common side effects were anorexia, nausea, weight loss, dizziness, and nervousness. Three patients developed suicidality with no completed suicide and one patient developed an episode of hypomania with venlafaxine [21].

10. Buspirone

In an open-label study of treatment of 25 subjects with pediatric social phobia, buspirone (mean optimal dose was 28 mg daily) showed some efficacy in the treatment of the phobia. Only three children improved enough to continue the medication after study completion (nine weeks) [22].

Generalized anxiety disorder

1. Sertraline

Ryan et al. evaluated the efficacy of sertraline (n=11) compared to placebo (n=11) in children and adolescents (ages 5-17 years with mean age 11.7+3.9 years) with GAD in a 9-week long, double-blind RCT. Primary outcome measures were evaluated by the HAM, and the CGI-I and -S scales. Sertraline was started at 25 mg/day, and then it was titrated to 50 mg/day from week two to nine. The HAM total, psychic and somatic factor scores showed a significant benefit for sertraline from week four to the end of the study. About 90% of patients taking sertraline improved on the CGI scale compared to 10% in placebo. However, only two patients taking sertraline markedly improved. Dry mouth, drowsiness, leg spasm, and restlessness were the adverse effects common in the sertraline group while dizziness, nausea, and stomach pain were observed in the placebo group [23].

2. Duloxetine

This 10-week long double-blind RCT of duloxetine (n=135, dose 30-120 mg) and placebo (n=137) was followed by an 18-week open-label phase (dose 30-120 mg). This study included children and adolescents (ages 7-17 years) with GAD who received duloxetine or placebo in a flexible-dose manner depending on the tolerability and response. The mean daily dose was 53.6 mg during the initial phase, followed by 66 mg (continued on duloxetine) and 60.4 mg (transition from placebo) during the open-label phase. Efficacy measures included the Pediatric Anxiety Rating Scale (PARS), CGI-S, and Children’s Global Assessment Scale (CGAS), while safety was assessed by the Columbia Suicide Severity Rating Scale (C-SSRS). The duloxetine group showed significant improvement in PARS, CGI-Severity, and CGAS in terms of symptomatic response, remission and functional remission (CGAS >70% improvement). The mean improvement was statistically significant in the duloxetine group, while the placebo group showed significant mean improvement in adolescents. During the second phase, there was a significant mean improvement in GAD symptoms in the group of duloxetine who transitioned from placebo.

Severe adverse events in the acute phase included suicidal ideation and self-injurious behaviors in one subject with a history of depression and suicidal ideations. In the open-label phase, the duloxetine group manifested acute psychosis, bipolar disorder, suicidal ideations, tonsillitis, and adenoiditis. A patient who suffered from apathy and later developed suicidal in the tapering phase was eliminated from the study. In the acute phase, seven patients in duloxetine and six in placebo discontinued due to the adverse events which include nausea. Duloxetine group exhibited side effects in a 28-week period resulting in discontinuation of the treatment included nausea, suicidal ideation, vomiting, abdominal pain, upper dyspepsia, self-harming thoughts, bipolar disorder, abnormal behavior, irritability, fatigue, initial insomnia, somnolence, apathy, vaginal bleeding, blepharospasm, pharyngeal inflammation, and severe adenoiditis. When duloxetine was tapered, headache and upper abdominal pain were observed. Duloxetine caused a three-fold increase in alanine transaminase which became normal after discontinuing it.

During the open-label phase, suicidal ideations with uncompleted suicidal attempts were reported seen in two patients. In comparison to baseline (end of the acute trial), three patients in each group (receiving duloxetine from the start and one switched from placebo to duloxetine) manifested treatment-emergent suicidal ideations. Duloxetine group exhibited more adverse events like nausea, vomiting, decreased appetite, oropharyngeal pain, cough, dizziness, and palpitations. In the acute phase, duloxetine showed a greater increase in pulse rate than the placebo group; also weight loss was seen in the duloxetine group, while weight gain was observed in the placebo group. In the second phase, the duloxetine group (receiving duloxetine from the beginning) showed a decrease in systolic and diastolic blood pressure, pulse, and increase in weight. The other group, which shifted from placebo to duloxetine, showed a mild increase in pulse, blood pressure, and weight [24].

3. Venlafaxine ER

Rynn et al. reported the results of two RCTs of eight weeks (each and tapering period of 14 days) evaluating the efficacy of venlafaxine ER (n=157) compared placebo (n=163) in children and adolescents of age 6-17 years with GAD [23]. The primary outcome was evaluated by the composite score of nine delineated items of GAD section of a modified version of the Schedule for Affective Disorders and Schizophrenia for School-Age Children (Columbia K-SADS). Primary efficacy was determined by the change in baseline to endpoint score. Overall score on the nine delineated items, PARS, Hamilton Anxiety Rating Scale (HARS), Screen for Child Anxiety Related Emotional Disorders, and CGI scores were evaluated for secondary outcomes. The response was defined as a 50 % decrease in Columbia K-SADS GAD section and A score of <3 on CGI. There was a difference in the results of the two studies with one showing significant improvements in primary (decrease of 17.4 points with venlafaxine vs 12.7 points placebo on Columbia K-SADS) and secondary outcomes in the venlafaxine group, while the other showed improvement in secondary outcomes but not primary ones in venlafaxine group. The severity component of the GAD section of Columbia K-SADS showed significant improvement in the venlafaxine group compared to placebo in both studies, while an improvement on impairment component was significant with venlafaxine in one study. Secondary outcomes favored venlafaxine in the first study, while the second study showed as significant between-group differences only with respect to CGI severity of illness and improvement scores. Both studies showed significant between-group differences in CGI score. CGI suggested improvement with venlafaxine( 69%) compared to placebo (48%).

Asthenia, anorexia, pain, and somnolence were common adverse effects in the venlafaxine group, twice as high as for placebo. Somnolence was common among adolescents. Changes in height, weight, pulse rate, blood pressure, and cholesterol level were significant with venlafaxine. Initially, 25% of placebo and 24% of the venlafaxine group dropped prematurely. Two subjects in the venlafaxine group discontinued treatment due to aggressive behaviors. On subgroup analysis, primary outcome measure improvement from baseline was observed both in children and adolescents. No gender difference in baseline severity existed. Tapering-related adverse events were 32% in the venlafaxine group and 16.5 % in the placebo group with dizziness more common with venlafaxine. Serious adverse events were observed in the first study with two participants dropping out of the study: one boy had suicidal ideation 48 hours after the last full dose, and one girl in the placebo group attempted suicide. In the second study, a girl showed agitation, and confusion resulted after venlafaxine taper. EKG changes like abnormal right atrial rhythm with short PR interval; and left atrial rhythm, rightward axis, and non-specific ST abnormality were observed in the venlafaxine group. These EKG changes were not significant enough to cause the withdrawal of participants.

4. Alprazolam

In an RCT of 30 children with the overanxious disorder (n=21) and avoidant disorder (n=9) one week of placebo administration was followed by either placebo or alprazolam. The medication was tapered with placebo substitution in week five, and all the subjects received placebo on week six. There was a strong treatment effect in both the alprazolam and placebo group. Alprazolam was superior to placebo only on clinical global ratings, and the difference was not statistically significant [25].

Panic disorder

1. Paroxetine

In a retrospective chart review of 18 participants ages 7-14 years (12 male and 6 female), Masi et al. reviewed the efficacy and safety of paroxetine for panic disorder. Participants with medical causes of panic disorder were excluded from the study. Paroxetine was started with an initial minimal dose of 8.9± 2.1 mg/day and was gradually titrated up to 40 mg/day depending upon clinical response and tolerability. CGI-I scale was used to assess clinical response among participants and adverse effects were monitored retrospectively at each follow-up visit. These patients were followed for a period of 11.7± 8.3 months (range 2-24 months). About 83.3% (15 out of 18 patients) reported an improvement in symptoms of panic disorder on the CGI scale (scores of ≤ 2). It took 21.87± 7.1 days (range 10-30 days) for these participants to respond, with the average dose at the initial response of 22.00± 7.75 mg/day. The average dose for participants with a response was 22.67± 9.61 mg/day (range 10-40 mg) at the end of the study. Paroxetine was well-tolerated with mild and transient side effects. The most common adverse effects were nausea, tension, agitation, sedation, insomnia, palpitations, and headache [26].

2. Clonazepam

Biederman investigated the efficacy of clonazepam for anxiety disorder with panic-like symptoms among three pre-pubertal children [27]. Two of these children were 11 years old, and one was 8 years old. These patients were prescribed 0.5-3 mg daily dose of clonazepam. These patients reported an improvement in their anxiety symptoms during a follow-up period of five months to three years without any side effects. Given the small sample size, the results of this case series should be carefully interpreted [27].

Separation anxiety disorder

1. Imipramine

In the second phase of a six-week-long double-blind RCT, children (n=21) with age range of 6-11 years (mean age 9.5±0.8 years) were assigned to either imipramine (n=11) or placebo (n=10) group. The participants were diagnosed with a separation anxiety disorder who failed to respond to an initial psychotherapeutic intervention. Of these, only half of the subjects improved. The imipramine was started at 25 mg/ day for three days, after which it was increased to 50 mg for next four days, and subsequently increased gradually with the maximum dose of 5 mg/kg/day. By the end of the study, the dose of imipramine was 75-275 mg /day with a mean dose of 153 mg/ day (4.67 mg/kg). EKG was obtained at the initial visit, and then with each dose increment after the first increase to 50 mg, and no significant changes were observed. Pre-study assessments included the Wechsler Intelligence Scale for Children-Revised, Diagnostic Interview Schedule, problem list, Conner’s Questionnaire by parents and teachers, and Children’s Manifest Anxiety Scale for parents and youth. The global rating of improvement, which was rated by the child psychiatrists, parents, and teachers, was also performed. The assessment measures performed by parents, teachers and children self-rating failed to show any significant differences between the two groups. About 40-60 % of subjects improved overall without any difference between the two groups. Side effects were reported by 8/11 subjects in the imipramine group and 2/9 in the placebo group. Imipramine group had 12 moderate to severe complaints out of 18 total complaints, with the irritability and anger most frequent side effect. None of the side effects were serious enough to warrant a dropout [28].

2. Clonazepam

In a double-blind cross-over study of 15 patients with separation anxiety disorder, subjects were treated with clonazepam (up to 2 mg /day ) and placebo. There were no significant treatment differences between the two groups. The authors did not support the use of clonazepam for separation anxiety disorder [29].

Discussion

Current literature suggests a role of pharmacotherapy in the management of moderate to severe anxiety. An SSRI is considered as the first-line of treatment with an option of switching to another SSRI or Serotonin and norepinephrine reuptake inhibitors (SNRIs) in patients with persistent anxiety [30]. However, the dose of SSRI should be optimized as the first step, since the improvement is dose-related and is dependent on serotonergic activity. Pediatric anxiety disorders respond to antidepressants early in the course of treatment with greater effect size and quicker response for SSRIs compared to SNRIs [31]. Functional improvement should be considered to be an important indicator determining the duration of pharmacotherapy [32]. The side effects including headache, gastric distress, insomnia, fatigue, and sedation should be closely observed. The patient should be advised about the transient and milder nature of these side effects. Suicidal ideations are a serious but less frequent side effect. Behavioral activation can be a distressing side effect of antidepressants, leading to the discontinuation of antidepressants [20]. To access the efficacy and monitoring for the adverse effects regular visits are advised. The most common reasons for treatment failure are poor compliance, inadequate medication dosing, and inadequate duration of the medication trial. Pediatric anxiety disorders are often associated with poor psychosocial functioning, academic underachievement, learning difficulties, substance abuse, relationship problems, and suicide attempts. Psychotherapy, particularly CBT as a stand-alone treatment and medications are effective for various anxiety disorders [11]. Depending on the severity and chronicity of the illness, the presence of psychosocial stressors, comorbid illnesses, and level of functioning, clinicians can choose the treatment options for anxiety disorders.

## Conclusions

A complete bio-psycho-social assessment, a working diagnosis, physical examination, and a comprehensive treatment plan that includes tangible goals is necessary before starting medications for anxiety disorders. SSRIs are the most common and efficacious treatment for anxiety disorders in youth. A combination of psychotherapy and medications usually results in better treatment outcomes in several anxiety disorders.
